# Knowledge as a key determinant of public support for autonomous vehicles

**DOI:** 10.1038/s41598-024-52103-6

**Published:** 2024-01-25

**Authors:** Hao Tan, Jiayan Liu, Cong Chen, Xue Zhao, Jialuo Yang, Chao Tang

**Affiliations:** State Key Laboratory of Advanced Design and Manufacturing Technology for Vehicle, Hunan University, Changsha, China

**Keywords:** Human behaviour, Psychology and behaviour

## Abstract

Autonomous vehicles (AVs) have the potential to revolutionize transportation safety and mobility, but many people are still concerned about the safety of AVs and hesitate to use them. Here we survey 4112 individuals to explore the relationship between knowledge and public support for AVs. We find that AV support has a positive relationship with scientific literacy (objective knowledge about science) and perceived understanding of AV (self-assessed knowledge). Respondents who are supportive of AVs tended to have more objective AV knowledge (objective knowledge about AVs). Moreover, the results of further experiments show that increasing people's self-assessed knowledge or gaining additional objective AV knowledge may contribute to increasing their AV support. These findings therefore improve the understanding of the relationship between public knowledge levels and AV support, enabling policy-makers to develop better strategies for raising AV support, specifically, by considering the role of knowledge, which in turn may influence public behavioural intentions and lead to higher levels of AV acceptance.

## Introduction

Autonomous vehicles (AVs) are regarded as emerging and disruptive technologies, with the potential to increase traffic efficiency^1,2^, reduce pollution^3,4^, reduce traffic accidents and save millions of lives^5–10^. Many countries are working to advance the development of AVs and focusing on making AVs socially acceptable^11,12^. However, many people worry about the safety of AVs and are hesitant to use them^13–15^. It is therefore important to explore the factors that influence public support for AVs (AV support).

Public attitudes are decisive factors for the development and deployment of new technologies, and understanding what factors influence public attitudes toward AVs is an active research topic^16^. Factors affecting AV attitudes are thought to fall into two levels^17^. Micro-level concerns socio-demographics (e.g., age, gender and education)^18–21^, personality (e.g., technology savviness and trust)^22,23^ and travel behavior (e.g., individuals’ access to mobility and driving experience)^24,25^. Meso-level concerns exposure to AVs (e.g., knowledge and experience)^26,27^, domain-specific system evaluation (e.g., perceived usefulness and safety)^28,29^, symbolic-affective aspects (e.g., hedonic motivation and subjective norm)^20,30^, and moral-normative aspects (e.g., perception of risks and benefits)^31^.

Knowledge is an important construct in understanding public attitude and behaviour^32–37^, and the results of a study suggest that the most unfavourable views of fully autonomous vehicles are held by the least knowledgeable consumers^38^. Two knowledge constructs have been distinguished, the first is objective knowledge (accurate information about the product stored in long-term memory), and the second is self-assessed knowledge or subjective knowledge (people’s perceptions of what or how much they know about a product)^39^.

In the AV field, the results of some studies suggest a significant positive trend between knowledge and attitude^21,40^; others suggest that knowing more about AVs is associated with more negative attitudes toward them^21,41–43^. In addition, self-assessed knowledge and objective knowledge have been distinguished by some studies, which showed that they were both related to acceptance, confidence, and behavioural intention of AV^38,44–46^. However, there is limited research on the relationship between public support for AV and knowledge.

Support refers to the way in which a person evaluatively orients himself to some object through either his attitudes or his behavior^47^. Specific support is directed to the perceived decisions, policies, actions, utterances or the general style of the specific object. Autonomous vehicles, climate change, vaccines, nuclear energy, and genetically modified food are often widely discussed as scientific issues^48,49^. Although the findings of studies suggest that public support toward these scientific issues are often associated with knowledge^33,50,51^, the influence of knowledge on public support cannot be broadly extended from one application to any other^34^.

Therefore, we explore the relationship between knowledge and AV support in terms of both self-assessed knowledge and objective knowledge in China, one of the most populous countries globally^52^, which could become the largest and most challenging autonomous driving market^53,54^. The self-assessed knowledge we examined in this study refers to the extent to which a person thinks he/she knows about AVs. Objective knowledge in our study includes scientific literacy (objective knowledge about science) and objective knowledge about AVs (objective AV knowledge). Scientific literacy measure refers to the public's knowledge of physics and earth sciences, astronomy, biologic sciences and human origins, health care, and AV, and is widely used in national public knowledge surveys^35,55^. According to the National Science Foundation's measure of the public's scientific literacy^35,56^, objective AV knowledge includes understanding the definitions of AV, understanding AV-related concepts (e.g., terms such as degree of automation, connected vehicles, etc.), and understanding of some public policy issues or social conditions that involve or directly affect AV. AV support in our study refers to public attitudes toward AVs in general^51^, including support for action toward AVs^48^.

We have conducted a series of surveys and experiments with a total of 4122 participants (the number of participants in each study can be found in Table 1). These studies encompassed a combination of online surveys and in-person experiments. Self-assessed knowledge and objective knowledge of science and technologies are always researchers’ focus^57^. Previous studies have found that self-assessed knowledge, objective knowledge, and public attitudes toward AVs are significantly related^38,45^. With this as backdrop, the relationship between self-assessed knowledge, scientific literacy (objective knowledge about science) and general public support for AV is explored in Study 1. There is a wide range of opinions about AVs^58^. The previous research have revealed that while people are willing to ride in an AV, most are not ready to buy one^40^. Therefore, we added questions about willingness to ride and buy AVs to public support measure in study 2a based on study 1^31,45,59,60^. When considering different scenarios, public attitudes will also be different^61^. Regarding AV issues, it is necessary to consider the road users in different scenarios, such as drivers, passengers, or pedestrians, because they hold different perspectives and bear various risks of traffic accidents^62,63^. Based on these research results, we have conducted an in-depth exploration of the relationship between respondents’ knowledge and support across three different scenarios (riding in AVs by themselves; family or friends riding in AVs; and AVs driving on the open road) in Study 2b. Furthermore, Study 3a and Study 3b use interventions to assess whether changing participants’ self-assessed knowledge or gaining additional objective AV knowledge through education could lead to changes in public support. This is an extended application of the "knowledge deficit model" in the field of AV, which proposes that more information increases public knowledge levels about a given topic and thus leads to improved attitudes and practice^64–66^. Accordingly, we put forward the following hypotheses:

**Table 1:** Characteristics of respondents.

		Study 1	Study 2a	Study 2b	Study 3a	Study 3b
Gender	Male	50.4%	47.6%	49.7%	52.7%	53.2%
	Female	49.6%	52.1%	50.3%	47.3%	46.8%
Age	18–29	56.9%	63.3%	57.2%	60.2%	56.7%
	30–44	33.8%	29.4%	34.7%	15.7%	30.2%
	45–59	6.8%	5.9%	6.5%	21.3%	11.6%
	≥ 60	2.6%	1.4%	1.6%	2.8%	1.5%
Education	Middle school and below	9.1%	5.2%	7.3%	10.1%	5.9%
	High school	14.2%	13.0%	18.9%	12.1%	12.5%
	Junior college	16.7%	17.4%	18.9%	14.6%	10.1%
	Undergraduate	48.1%	51.5%	46.5%	37.1%	49.8%
	Graduate and above	12.0%	12.9%	8.3%	26.1%	21.7%
Monthly income (CNY)	< 2000	26.2%	26.2%	17.8%	35.6%	23.6%
	2000–5000	35.4%	33.8%	37.5%	25.8%	28.8%
	5000–10,000	26.0%	29.1%	35.4%	26.9%	30.2%
	> 10,000	12.3%	10.9%	9.3%	11.8%	17.3%
Driving experience	Yes	64.5%	59.9%	67.3%	62.7%	65.7%
	No	35.5%	40.1%	32.7%	37.3%	34.3%
N		1037	915	995	357	808

The sample distribution in each study is reported by percentage.

### Hypothesis 1

People’s perceived understanding of AVs (self-assessed knowledge) and scientific literacy (objective knowledge about science) has a positive relationship with AV support (general support).

### Hypothesis 2

People’s self-assessed knowledge and objective AV knowledge (objective knowledge about AVs) has a positive relationship with AV support (including general support, willingness to ride in an AV, and willingness to buy an AV).

### Hypothesis 3

People’s self-assessed knowledge and objective AV knowledge has a positive relationship with AV support under three scenarios (yourself riding in an AV; relatives or friends riding in an AV; AVs driving on the open road).

### Hypothesis 4

Increasing people’s self-assessed knowledge would have a positive effect on AV support.

### Hypothesis 5

Gaining additional objective AV knowledge would have a positive effect on AV support.

## Methods

### Ethics statement

This study was approved by the Research Ethics Committee of Hunan University (No. 2019001). Oral consent was obtained from the respondents, and they were assured that all the results would be disseminated in aggregate form to guarantee anonymity and confidentiality. We confirm that all research was performed in accordance with relevant guidelines/regulations, and informed consent was obtained from all participants and/or their legal guardians.

### Data

Online respondents were recruited through the Baidu Data Crowdsourcing Platform, which has more than 17,000,000 respondents in its sample database and covers 300 cities in China. Quality control of the questionnaire was done by checking the time of participation, and 62 non-compliant participants were excluded, which resulted in a total of 4112 participants included in our final analysis. Analysis was performed using 3755 eligible questionnaire data points from the online survey (Studies 1, 2a, 2b and 3b). Due to the experimental design, Study 3a took the form of an offline experiment. The experiment was conducted by trained researchers on the experimental team for respondent recruitment and interviews across China, and 357 participants took part in the offline experiment.

### Study 1

We aimed to explore the relationship between the public’s self-assessed knowledge, scientific literacy (objective knowledge about science) and their AV support in Study 1. First, we asked the respondents to evaluate their AV support (“1 = not support at all” and “5 = very support”) and self-assessed knowledge (“1 = not understand at all” and “5 = understand very well”) using a five-point Likert scale. After completing the assessment, 15 true–false questions measured respondents’ scientific literacy score, which was calculated with the results of their answers (1 point for the correct answer, 0 points for the wrong answer, or “Do not know”). These questions were derived from previous studies on public knowledge^10,35,55^, including a question about AVs^67^. For robustness, we also replicated the analyses after removing the AV question from the “scientific literacy” scale. The result of the sensitivity analysis was shown in supplementary file S1, which indicated that our results are robust. Finally, we asked the respondents for their basic personal information, including their gender, age, income, education, purchasing habits, and driving experience.

### Study 2a

We aimed to explore the relationship between knowledge (including self-assessed knowledge and objective AV knowledge) and willingness to ride in or buy AVs in Study 2a. Therefore, in addition to the “Support for AVs” section of the questionnaire, questions about riding willingness and purchasing willingness (“1 = certainly would not” and “7 = certainly would”) were added based on Study 1. We also changed the original 15 questions about science to 10 questions related to AVs, which were adapted from previous studies in the AV field^10,67–71^.

### Study 2b

Study 2b used the “situation introduction” method to ask respondents to imagine themselves in three different scenarios. The purpose was to explore the AV support from the perspective of varying risk holders. Therefore, three scenarios were added to the “Support for AVs” part in a questionnaire, and the respondents were asked about their AV support in these scenarios (“1 = not support at all” and “9 = very support”). The descriptions of these scenarios were as follows: “Please imagine yourself riding in AVs in the future”; “Please imagine your relatives or friends riding in AVs in the future”; and “Please imagine AVs driving on the road in the future.”

### Study 3a

We aimed to explore how changes in respondents’ self-assessed knowledge affect their AV support through experiments in Study 3a. Similar to previous studies, we first asked respondents to evaluate their support for and understanding of AVs on a 100-point scale. The aim of this measurement change was to analyse respondents’ change in support and self-assessed understanding after self-assessed knowledge changes. Subsequently, we measured respondents’ objective AV knowledge using the same 10 questions as Study 2. People’s self-assessed knowledge can be changed when encouraging them to try to generate explanations or giving them reference points^72–74^. Based on this, we attempted to provide participants with reference points by showing them their score on the objective knowledge test, thereby changing their self-assessed knowledge. Subsequently, respondents were asked to evaluate how much they knew about AVs again. Finally, the respondents were asked to choose a value from 1 to 100 to assess their AV support and to enter their basic personal information.

### Study 3b

In Study 3b, we used the same research strategy to explore how gaining additional objective AV knowledge affects support. This study’s process was similar to that of Study 3a, except that after collecting the initial data of respondents’ knowledge and support, respondents were asked to read three paragraphs of text about AVs and then the changes in their support were collected. Based on the results in previous research, the text was divided according to three aspects, research history, concept, and definition, as well as relevant laws and regulations. After their reading, the respondents were asked to answer three questions based on what they had read to measure the additional objective AV knowledge they gained.

### Data analysis

Our analysis quantified the relationship among objective knowledge, self-assessed knowledge, and AV support. SPSS was used for data analysis. Unless otherwise stated, all regression coefficients were conducted by ordinary linear regression analysis, and all test statistics were two-sided.

We conducted linear regression analyses on the relationship between knowledge and support. In Studies 1, 2a, and 2b, we directly assessed the questionnaire results of respondents’ self-assessed and objective knowledge scores and support as independent variables and dependent variables, respectively. In Studies 3a and 3b, we conducted difference coding by calculating the difference between self-assessed knowledge and support before and after experiments. In Study 3b, we regarded the post-experimental knowledge score as the gained objective knowledge about AV.

To compare the differences between participants’ objective knowledge in the different groups, we created a dummy variable, coded as 1 or 0 to represent the different support levels. We chose the support group as the reference for comparison with the other remaining groups (see Table S3 for details).

Subsequently, in Studies 2a and 2b, the differences in respondents’ objective AV knowledge with regard to their different support levels were calculated by an independent sample *t* test conducted using SPSS. In Study 1, we categorized respondents as 'supporters' if they rated their support for autonomous vehicles (AVs) from 1 to 2, as 'nonsupporters' if they rated their support from 4 to 5, and as 'neither' if they selected a 3. In Study 2a, public support was measured by 3 items using 7-point Likert scales ("1 = not support at all" and "7 = strongly support"). We divided the mean of participants' support rating responses into 7 equal parts, resulting in a final formation into 7 levels. Respondents were classified as "nonsupport for AV" if their AV support level was less than 3 (including 3), as "support for AV" if the value was greater than 5 (including 5), and as "neither" including only level 4. In Study 2b, the respondents were asked about their AV support from three scenarios (“1 = not support at all” and “9 = strongly support”). In each scenario, they were classified as "nonsupport for AV" if they responded between 1 (not support at all) and 4, as "support for AV" if they responded between 6 and 9 (strongly support), and as "neither" if they chose the midpoint response of 5 (neither nonsupport nor support). The consistency of the two groups’ variance was represented by the variance difference test’s significance, indicating whether the data qualified sufficiently. After verifying a nonsignificant difference in the variance, the difference test was performed on the two groups’ mean knowledge score. The significance of the test results was then used to judge whether there was a significant difference in the means of different groups.

### Ethics approval

The research study was approved by the Commission for Ethics in Research of Hunan University. Participation was voluntary and all respondents provided their consent to participate in the survey.

## Results

A series of questionnaire surveys were administered to 4112 Chinese adults to identify the relationship between the Chinese public’s knowledge of and AV support. The study was divided into four parts. There was no participant overlap across studies, ensuring that each individual contributed to only one of the studies. Table 1 reports the demographic information regarding this series of surveys, mainly respondents’ gender, age, education, monthly income, and driving experience (Table S1 lists exact sample details).

Study 1 investigated 1037 Chinese adults (49.6% female) to explore the relationship between the public’s self-assessed and general objective AV knowledge and their AV support.

**Figure 1:**
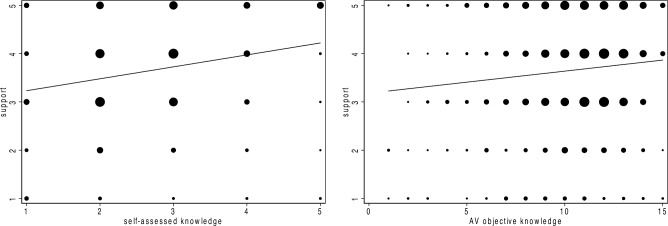
Predicted relationships between support and knowledge. The effect based on linear regression analysis applied in Study 1 on 1037 individuals; shading represents the 95% CI; marker sizes are proportional to joint frequency.

Respondents were asked to evaluate their support for AVs using a 5-point Likert scale (57.5% support, 15.1% nonsupport). Respondents’ self-assessed knowledge was measured by a 5-point Likert scale. Furthermore, scientific literacy (objective knowledge about science) was measured by 15 scientific true or false judgement questions (Answered "I don't know": 13.5%; Answered all questions correctly: 2.5%). The average score of respondents’ scientific literacy was 10.33 (Physics and earth science: mean = 3.94, SE = 0.043; Astronomy: mean = 1.61, SE = 0.018; Biological science and human origins: mean = 2.22, SE = 0.025; Health care: mean = 2.09, SE = 0.024; AV: mean = 0.46, SE = 0.015). Figure 1 shows that as scientific literacy increases, AV support increases (*B*(unstandardized coefficient) = 0.046, *β*(standardized coefficient) = 0.109, *t* = 3.538, *P* < 0.0001, 95% confidence interval (CI) (0.020, 0.071), *R*^*2*^ = 0.012). Furthermore, as self-assessed knowledge increases, support increases (*B* = 0.247, *β* = 0.234, *t* = 7.759, *P* < 0.0001, 95% confidence interval (CI) (0.185, 0.310), *R*^*2*^ = 0.055; Table S2 lists the details of all the studies). The above results indicate that hypothesis 1 has been verified.

In Study 2a, support was measured by averaging three items, including a direct measure of support level of AVs, willingness to ride in and willingness to buy AVs, and the correlation between these three items was 0.494, 0.471,0.659 (*P* < 0.01). Study 2a (*N* = 915; 52.1% female) reached the same conclusion as Study 1 through regression analysis, which showed that the higher respondents’ self-assessed knowledge score, the more supportive they are of AVs (*B* = 0.277, *β* = 0.312, *t* = 9.922, *P* < 0.0001, 95% confidence interval (CI) (0.222, 0.331), *R*^*2*^ = 0.097). For objective knowledge (Answered "I don't know": 22.9%; Answered all questions correctly: 0.7%), this trend still exists, but the significance decreases (*B* = 0.073, *β* = 0.095, *t* = 2.886, *P* < 0.05, 95% confidence interval (CI) (0.023, 0.123), *R*^*2*^ = 0.009). The above results indicate that hypothesis 2 has been verified.

**Figure 2:**
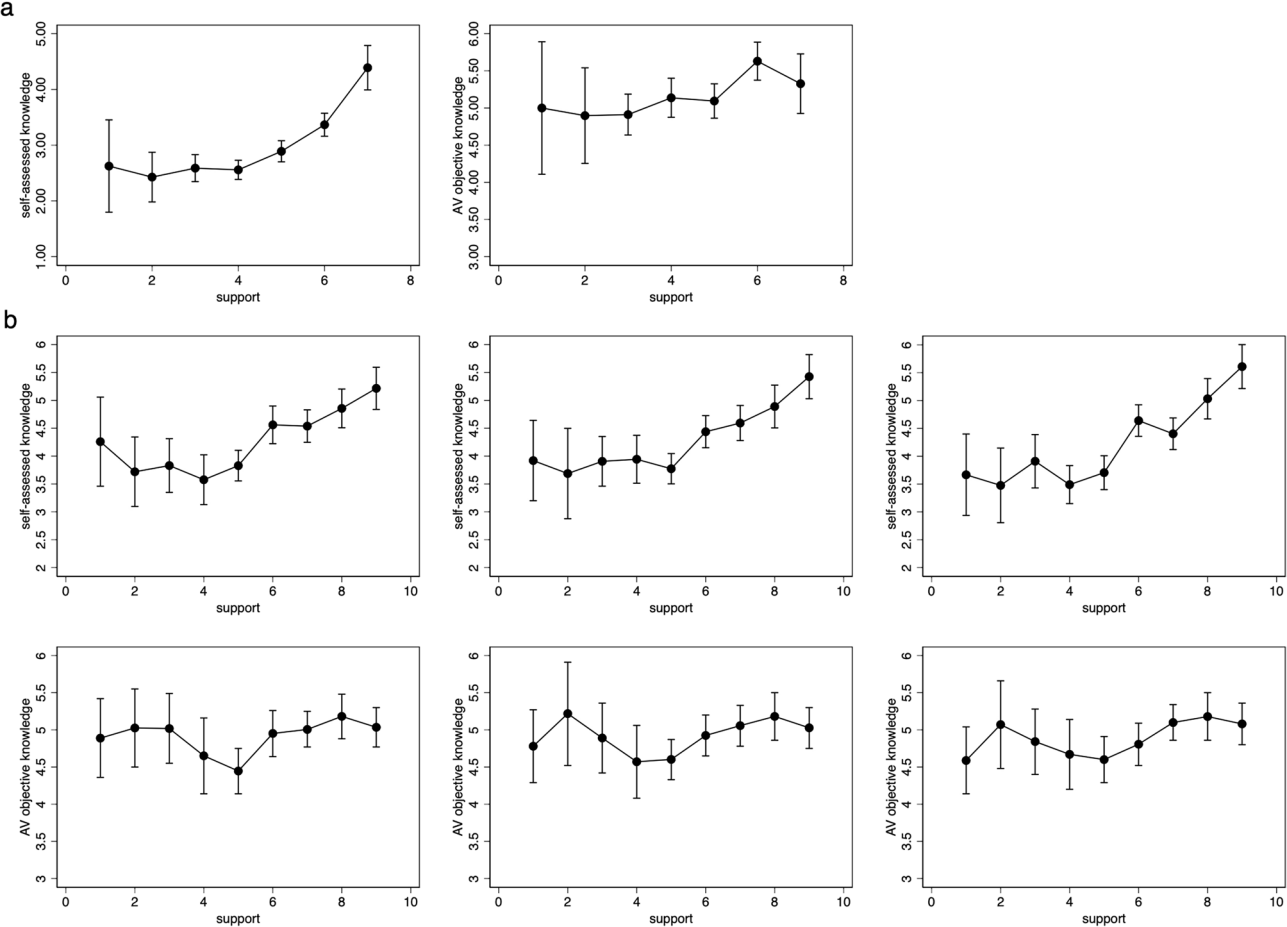
Objective and self-assessed knowledge means by support. Respondents were asked to report their AV support and self-assessed and objective AV knowledge. (**a**) Shows the results in Study 2a, and (**b**) indicates the results in Study 2b.

Since the significance of the relationship between objective AV knowledge and support decreased compared to Study 1, we then conducted a further analysis to examine respondents’ objective AV knowledge with regard to their different support levels, and these results showed that respondents who support for AVs had, on average, the most objective AV knowledge. As shown in Fig. 2a, the comparison of the average objective knowledge of respondents with their different levels of support (support, nonsupport, or neutral) shows that the objective knowledge score of respondents who support AVs is higher than that of non-supporters (*P* < 0.01), however, not significantly different from those who were neutral about AVs (*P* = 0.059) (Table 2).

**Table 2:** Differences in mean objective knowledge score on AVs by different support levels.

	Support	Neither	Nonsupport
Mean OKS	5.33 (1.869)^1^	5.14 (1.904)	4.92 (1.652)
Support	–	0.197^2^ (0.155)^3^	0.418** (0.148)
Neither	0.197 (0.155)	–	–
Nonsupport	0.418** (0.148)	–	–

The differences in Study 2a (this table) and Study 2b (Table 3) were calculated by an independent sample *t* test in SPSS.

*OKS* objective knowledge score.

^1^Standard deviations.

^2^Mean difference of objective knowledge scores between two groups (the difference is calculated as the score of the support group minus the score of the nonsupport/neither groups).

^3^Standard error difference.

*P < 0.05; **P < 0.01; ***P < 0.001.

We assessed the respondents’ AV support in three different scenarios in Study 2b (riding in AVs by themselves: 64.5% support, 20.4% nonsupport; family or friends riding in AVs: 61.7% support, 21.6% nonsupport; AVs driving on the open road: 63.1% support, 22.8% nonsupport). The data collection method was the same as that in the previous study (*N* = 995; 50.3% female).

Regression analysis results show that even under different scenarios, there is still a significant relationship in terms of “The higher respondents’ self-assessed knowledge score, the more supportive they for are of AVs” (riding in AVs by themselves: *B* = 0.221, *β* = 0.213, *t* = 6.868, *P* < 0.0001, 95% confidence interval (CI) (0.158, 0.285), *R*^*2*^ = 0.045; family or friends riding in AVs: *B* = 0.248, *β* = 0.240, *t* = 7.799, *P* < 0.0001, 95% confidence interval (CI) (0.186, 0.310), *R*^*2*^ = 0.058; AVs driving on the open road in the future: *B* = 0.304, *β* = 0.293, *t* = 9.651, *P* < 0.0001), 95% confidence interval (CI) (0.242, 0.366), *R*^*2*^ = 0.086). For objective AV knowledge. (Answered "I don't know": 21.9%; Answered all questions correctly: 0.7%), the effect of “support increases with objective knowledge” remained for the scenario of AVs driving on open roads (*B* = 0.110, *β* = 0.121, *t* = 2.771, *P* < 0.05, 95% confidence interval (CI) (0.032, 0.188), *R*^*2*^ = 0.008). However, these results are not statistically significant in the scenario of “riding in AVs by themselves” and “family or friends riding in AVs”. Detailed results are shown in Table S2b of the supplementary document.

Similar to the results of Study 2a, the results of comparing the average objective knowledge score of respondents with different support levels (support, nonsupport, or neutral) show that in all three scenarios, supporters had on average the most objective knowledge, with significantly more than that of neutral respondents (Fig. 2b). The independent sample *t* test verified the difference in the average knowledge score in the three scenarios (Table 3), which was somewhat similar with the conclusions of Study 2a. The findings of Study 2b partially support hypotheses 3, as it was found that AV support was positively correlated with self-assessed knowledge in three scenarios, but not with objective AV knowledge in the scenario of “riding in AVs by themselves” and “family or friends riding in AVs”. Also, we found that the objective knowledge between AV supporters and neutrals showed significant differences, but no significant differences were found between the knowledge of AV non-supporters and the other two.

**Table 3:** Differences in mean objective knowledge score on AVs by different support levels across three different scenarios.

	Scenario 1			Scenario 2			Scenario 3		
	Support	Neither	Nonsupport	Support	Neither	Nonsupport	Support	Neither	Nonsupport
Mean OKS	5.04 (1.777)	4.45 (1.870)	4.87 (1.826)	5.04 (1.781)	4.59 (1.760)	4.81 (1.906)	5.05 (1.772)	4.59 (1.880)	4.75 (1.847)
Support	–	0.579*** (0.163)	0.171 (0.144)	–	0.450** (0.156)	0.234 (0.144)	–	0.461** (0.168)	0.297* (0.139)
Neither	0.579*** (0.163)	–	–	0.450** (0.156)	–	–	0.461** (0.168)	–	–
Non-support	0.171 (0.144)	–	–	0.234 (0.144)	–	–	0.297* (0.139)	–	–

The differences in Study 2a (Table 2) and Study 2b (this table) were calculated by an independent sample *t* test in SPSS.

*P < 0.05; **P < 0.01; ***P < 0.001.

In Study 3, we explored the feasibility of strategies that affect respondents’ AV support by changing their self-assessed knowledge and making them gain additional objective AV knowledge. Study 3 was divided into two parts: offline and online experiments. Based on Study 1 and Study 2, we attempted to change respondents' self-assessed knowledge by showing them their score on the objective knowledge test in Study 3a and to make them gain additional objective AV knowledge by having them read materials about AVs in the online experiment. We then observed how their AV support changed.

Through a two-month offline questionnaire and interviews (*N* = 357; 47.3% female), changes in their support were collected from the respondents after they were presented with their objective knowledge score on AVs, measured by a self-assessment scale, consistent with previous studies. The second self-assessment result of the respondents minus the first was the indicator of changes in self-assessed knowledge and support (35.6% increase, 24.4% decrease, 40% not change).

Regression analysis results show that the effect of the change in self-assessed knowledge was significant and positive in terms of the change in support (*B* = 0.112, *β* = 0.121, *t* = 2.301, *P* < 0.05, 95% confidence interval (CI) (0.016, 0.207), *R*^*2*^ = 0.015). In other words, increasing people's self-assessed knowledge may help increase their AV support. The above result indicates that hypothesis 4 has been verified.

In the online experiment in Study 3b (*N* = 808; 46.8% female), respondents' objective AV knowledge was gained by reading the focal material, and answer scores after the experiment was regarded as an indicator of gained objective AV knowledge (0 point:15.8%, 1 point:23.9%, 2 points:33.5%, 3 points:26.7%). Changes in support were measured in the same way as in the offline experiments (45.5% increase, 34.7% decrease, 19.8% no change).

**Figure 3:**
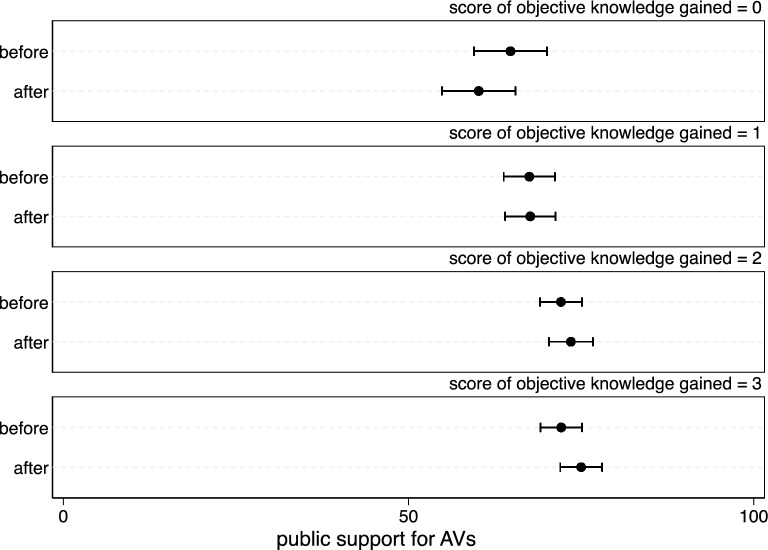
Differences in the mean level of support for AV by gaining additional objective AV knowledge. Respondents were asked about their level of support before and after experiment. As different levels of objective AV knowledge gained, the mean support levels before and after are both shown. The graph represents 95% confidence intervals (CIs).

Regression analysis of additional objective knowledge gained and the change in AV support shows that there is a significant positive correlation between the two variables (*B* = 2.234, *β* = 0.103,* t* = 2.940, *P* < 0.05, 95% confidence interval (CI) (0.742, 3.725), *R*^*2*^ = 0.011). As shown in Fig. 3, the more respondents' objective AV knowledge gained, the more supportive they are of AVs. Furthermore, this result confirms the feasibility of using conclusion 2 as a strategy to change the public’s AV support. Giving people access to more objective AV knowledge can increase their AV support, which indicates that hypothesis 5 has been verified.

## Discussion

Our results shed light on the role that knowledge (both self-assessed knowledge and objective knowledge) plays in AV support, and how changing self-assessed knowledge and acquiring objective AV knowledge impacts public support. We provide evidence showing that (1) participants’ AV support has a positive relationship with their perceived understanding of AV (self-assessed knowledge) and scientific literacy (objective knowledge about science), and (2) respondents who were supportive of AVs tended to have more objective AV knowledge. Moreover, (3) increasing people's self-assessed knowledge and gaining additional objective AV knowledge may both help increase their AV support. Although a few studies have attempted to understand the relationship between the knowledge and attitudes toward AVs^21,40^, this paper distinguishes between self-assessed and objective knowledge and contributes to the literature by providing some strategic support for improving public support.

Specifically, the more the respondents’ self-assessed knowledge and scientific literacy is, the more supportive they are of AVs, similar to results in many other studies^38,40,75^. In terms of objective AV knowledge, our results show that respondents who support for AVs have, on average, the most objective AV knowledge, with a significant difference between those who are unsupportive of AVs in Study2a, and those who are neutral in Study 2b. This may mean that when imagining specific scenarios (riding in AVs by themselves; family or friends riding in AVs; and AVs driving on the open road), respondents with the least AV knowledge are the least interested in the topic and therefore do not have an opinion about it^76^.

This study improves the understanding of the relationship between level of knowledge and AV support. Although place-specific differences exist, our study in China provides some strategic support for improving public attitudes towards AVs by changing their perceived understanding of AVs or gaining objective AV knowledge. For example, to improve support for autonomous vehicles, their perceived understanding of AV can be increased by increasing people's driving experiences^77–79^. Also, more information increases objective knowledge levels about AVs and thus leads to improved public support^64–66^. There are some limitations to this study that need to be discussed. The development of measures for the timely study of emerging phenomena such as automated vehicles do not always permit careful psychometric testing^38^, and there hasn’t been a universally-acknowledged scientific paradigm measuring objective knowledge to date^45^. Our measure of objective AV knowledge was not systematically assessed for validity and reliability. However, we believe that the measure has a high level of face validity. In addition, objective knowledge was significantly correlated with self-assessed knowledge^79–81^, and both indicators suggest that the higher knowledge people have, the more supportive they were for AVs. This convergence also seems to indicate that the findings involving objective knowledge of AV are valid and reliable. Another potential limitation of the study design is that responses might have been subject to order effects. The experiment of knowledge change in our study aimed at testing whether public support for AV would be affected. Therefore, these items were raised to respondents after the experiment. The initial measure of knowledge and AV support may have primed, anchored or otherwise influenced subsequent responses. However, the convergence of our post-experimental observations with the findings of Ranney et al. described above also seems to indicate that the findings involving the effect of knowledge change on support change are valid and reliable^82^. The questionnaire surveys were conducted only in China, and therefore, this study’s conclusions can only represent the relationship between AV support and knowledge level in the context of an East Asian culture. In addition, the online survey respondents may have been impacted by self-selection bias or the disproportionate youth of our samples. In addition to exploring the effects of AV support in China, as in this study, we look forward to further discussions with researchers from other cultural regions in the future. Furthermore, future research could explore how changes in knowledge can be used to increase AV support.

### Supplementary Information

Supplementary Information.

## Data Availability

All data needed to evaluate the conclusions in the paper are present in the paper and/or the Supplementary Materials S1. Additional data related to this paper may be requested from the authors.

## References

[1] Wang, J., Peeta, S. & He, X. Multiclass traffic assignment model for mixed traffic flow of human-driven vehicles and connected and autonomous vehicles. *Transp. Res. Part B Methodol.* **126** (2019).

[2] Hamadneh, J. & Esztergár-Kiss, D. Travel behavior of car travelers with the presence of park-and-ride facilities and autonomous vehicles. *Period. Polytech. Transp. Eng.* **50**, 101–110 (2022).

[3] Tate, L., Hochgreb, S., Hall, J. & Bassett, M. Energy efficiency of autonomous car powertrain. Report No. 0148-7191 (SAE Technical Paper, 2018).

[4] Vahidi A, Sciarretta A (2018). Energy saving potentials of connected and automated vehicles. Transp. Res. Part C Emerg. Technol..

[5] Waldrop MM (2015). No drivers required. Nature.

[6] LaFrance, A. Self-driving cars could save 300,000 lives per decade in America. *The Atlantic* **29** (2015).

[7] Bertoncello, M. & Wee, D. Ten ways autonomous driving could redefine the automotive world. *MCK* **6** (2015).

[8] Beza AD, Zefreh MM (2019). Potential effects of automated vehicles on road transportation: A literature review. Transpa. Telecommun..

[9] Tengilimoglu, O., Carsten, O. & Wadud, Z. Implications of automated vehicles for physical road environment: A comprehensive review. *Transp. Res. Part E Logist. Transp. Rev.* **169**, 102989 (2023).

[10] Talebpour A, Mahmassani HS (2016). Influence of connected and autonomous vehicles on traffic flow stability and throughput. Transp. Res. Part C Emerg. Technol..

[11] DOT, U. Automated Vehicles 3.0 Preparing for the Future of Transportation. (2018).

[12] Ki, J. A comparative analysis of autonomous vehicle policies among Korea, Japan, and France. (2020).

[13] Haboucha CJ, Ishaq R, Shiftan Y (2017). User preferences regarding autonomous vehicles. Transp. Res. Part C Emerging Technol..

[14] Schoettle, B. & Sivak, M. in *2014 International Conference on Connected Vehicles and Expo (ICCVE).* 687–692 (IEEE).

[15] Seapine Software. *Study finds 88 percent of adults would be worried about riding in a driverless car*, http://www.seapine.com/pr.php?id=217 (2014).

[16] Liu P, Xu Z, Zhao X (2019). Road tests of self-driving vehicles: Affective and cognitive pathways in acceptance formation. Transp. Res. Part A Policy Pract..

[17] Nordhoff S, Kyriakidis M, Van Arem B, Happee R (2019). A multi-level model on automated vehicle acceptance (MAVA): A review-based study. Theor. Issues Ergon. Sci..

[18] Bansal P, Kockelman KM (2017). Forecasting Americans’ long-term adoption of connected and autonomous vehicle technologies. Transp. Res. Part A Policy Pract..

[19] Hohenberger C, Spörrle M, Welpe IM (2016). How and why do men and women differ in their willingness to use automated cars? The influence of emotions across different age groups. Transp. Res. Part A Policy Pract..

[20] Acheampong RA, Cugurullo F (2019). Capturing the behavioural determinants behind the adoption of autonomous vehicles: Conceptual frameworks and measurement models to predict public transport, sharing and ownership trends of self-driving cars. Transp. Res. Part F Psychol. Behav..

[21] Charness N, Yoon JS, Souders D, Stothart C, Yehnert C (2018). Predictors of attitudes toward autonomous vehicles: The roles of age, gender, prior knowledge, and personality. Front. Psychol..

[22] Chen H-K, Yan D-W (2019). Interrelationships between influential factors and behavioral intention with regard to autonomous vehicles. Int. J. Sustainable Transp..

[23] Hegner SM, Beldad AD, Brunswick GJ (2019). In automatic we trust: Investigating the impact of trust, control, personality characteristics, and extrinsic and intrinsic motivations on the acceptance of autonomous vehicles. Int. J. Hum.-Comput. Interact..

[24] Bansal P, Kockelman KM, Singh A (2016). Assessing public opinions of and interest in new vehicle technologies: An Austin perspective. Transp. Res. Part C Emerging Technol..

[25] Nordhoff, S., De Winter, J., Kyriakidis, M., Van Arem, B. & Happee, R. Acceptance of driverless vehicles: Results from a large cross-national questionnaire study. *J. Adv. Transp.* (2018).

[26] Berliner RM, Hardman S, Tal G (2019). Uncovering early adopter’s perceptions and purchase intentions of automated vehicles: Insights from early adopters of electric vehicles in California. Transp. Res. Part F Psychol. Behav..

[27] Hartwich F, Witzlack C, Beggiato M, Krems JF (2019). The first impression counts–A combined driving simulator and test track study on the development of trust and acceptance of highly automated driving. Transp. Res. Part F Psychol. Behav..

[28] Zmud JP, Sener IN (2017). Towards an understanding of the travel behavior impact of autonomous vehicles. Transp. Res. Proc..

[29] Xu Z (2018). What drives people to accept automated vehicles? Findings from a field experiment. Transp. Res. Part C Emerg. Technol..

[30] Yuen KF, Chua G, Wang X, Ma F, Li KX (2020). Understanding public acceptance of autonomous vehicles using the theory of planned behaviour. Int. J. Environ. Res. Public Health.

[31] Liu H, Yang R, Wang L, Liu P (2019). Evaluating initial public acceptance of highly and fully autonomous vehicles. Int. J. Hum.-Comput. Interact..

[32] Shi J, Visschers VH, Siegrist M, Arvai J (2016). Knowledge as a driver of public perceptions about climate change reassessed. Nat. Clim. Change.

[33] Fernbach PM, Light N, Scott SE, Inbar Y, Rozin P (2019). Extreme opponents of genetically modified foods know the least but think they know the most. Nat. Hum. Behav..

[34] Mielby H, Sandøe P, Lassen J (2013). The role of scientific knowledge in shaping public attitudes to GM technologies. Public Underst. Sci..

[35] Durant JR, Evans GA, Thomas GP (1989). The public understanding of science. Nature.

[36] Gärling T, Evans GW (1991). Environment, cognition, and action: An integrated approach.

[37] Kaplan, S. Beyond rationality: Clarity-based decision making. *Environment, cognition, and action: An integrated approach*, 171–190 (1991).

[38] Sanbonmatsu DM, Strayer DL, Yu Z, Biondi F, Cooper JM (2018). Cognitive underpinnings of beliefs and confidence in beliefs about fully automated vehicles. Transp. Res. Part F Psychol. Behav..

[39] Park CW, Mothersbaugh DL, Feick L (1994). Consumer knowledge assessment. J. Consum. Res..

[40] König M, Neumayr L (2017). Users’ resistance towards radical innovations: The case of the self-driving car. Transp. Res. Part F Psychol. Behav..

[41] Liu P, Du M, Xu Z, Chu Y (2022). People with more misconceptions about automated vehicles might be more positive toward them. Transp. Res. Part F Psychol. Behav..

[42] Du M, Zhang T, Liu J, Xu Z, Liu P (2022). Rumors in the air? Exploring public misconceptions about automated vehicles. Transp. Res. Part A Policy Pract..

[43] Othman K (2023). Investigating how the public acceptance of autonomous vehicles evolve with the changes in the level of knowledge: A demographic analysis. Cogent Eng..

[44] Zhao, X., Yang, J. & Tan, H. in *International Conference on Human-Computer Interaction.* 297–308 (Springer).

[45] Tan H, Zhao X, Yang J (2022). Exploring the influence of anxiety, pleasure and subjective knowledge on public acceptance of fully autonomous vehicles. Comput. Hum. Behav..

[46] Keszey T (2020). Behavioural intention to use autonomous vehicles: Systematic review and empirical extension. Transp. Res. Part C Emerg. Technol..

[47] Easton D (1975). A re-assessment of the concept of political support. Br. J. Polit. Sci..

[48] Besley JC, Lee NM, Pressgrove G (2021). Reassessing the variables used to measure public perceptions of scientists. Sci. Commun..

[49] Peng Y (2020). The ideological divide in public perceptions of self-driving cars. Public Underst. Sci..

[50] Light, N., Fernbach, P. M., Rabb, N., Geana, M. V. & Sloman, S. A. Knowledge overconfidence is associated with anti-consensus views on controversial scientific issues. *Sci. Adv.* **8**, eabo0038 (2022). 10.1126/sciadv.abo0038 PMC9299547 35857847

[51] Jones, M. S., Delborne, J. A., Elsensohn, J., Mitchell, P. D. & Brown, Z. S. Does the US public support using gene drives in agriculture? And what do they want to know? *Sci. Adv.* **5**, eaau8462 (2019). 10.1126/sciadv.aau8462 PMC6739092 31535017

[52] Hertog, S., Gerland, P. & Wilmoth, J. India overtakes China as the world’s most populous country. (2023).

[53] Pizzuto, L., Thomas, C., Wang, A. & Wu, T. How China will help fuel the revolution in autonomous vehicles. *MCK* (2019).

[54] Lu, Y. *et al.* Forty years of reform and opening up: China’s progress toward a sustainable path. *Sci. Adv.* **5**, eaau9413 (2019). 10.1126/sciadv.aau9413 PMC6685713 31457075

[55] Zhang Z, Zhang J (1993). A survey of public scientific literacy in China. Public Underst. Sci..

[56] Arvizu, D. & Bowen, R. National Science Board. *Sci. Eng. Indic. 2014* (2014).

[57] Park CW, Lessig VP (1981). Familiarity and its impact on consumer decision biases and heuristics. J. Consum. Res..

[58] Bazilinskyy P, Kyriakidis M, de Winter J (2015). An international crowdsourcing study into people's statements on fully automated driving. Proc. Manuf..

[59] Zhang T (2019). The roles of initial trust and perceived risk in public’s acceptance of automated vehicles. Transp. Res. Part C Emerg. Technol..

[60] Liu P, Guo Q, Ren F, Wang L, Xu Z (2019). Willingness to pay for self-driving vehicles: Influences of demographic and psychological factors. Transp. Res. Part C Emerging Technol..

[61] Awad E (2018). The moral machine experiment. Nature.

[62] Shariff A, Bonnefon J-F, Rahwan I (2017). Psychological roadblocks to the adoption of self-driving vehicles. Nat. Hum. Behav..

[63] Bonnefon J-F, Shariff A, Rahwan I (2016). The social dilemma of autonomous vehicles. Science.

[64] Brown S (2009). The new deficit model. Nat. Nanotechnol..

[65] Gustafson A, Rice RE (2016). Cumulative advantage in sustainability communication: Unintended implications of the knowledge deficit model. Sci. Commun..

[66] Schultz, P. W. Knowledge, information, and household recycling: Examining the knowledge-deficit model of behavior change. In *New tools for environmental protection: Education, information, and voluntary measures* (2002).

[67] Shadrin, S. S. & Ivanova, A. A. Analytical review of standard Sae J3016 «taxonomy and definitions for terms related to driving automation systems for on-road motor vehicles» with latest updates. *Avtomobil'. Doroga. Infrastruktura.* **10** (2019).

[68] Lu N, Cheng N, Zhang N, Shen X, Mark JW (2014). Connected vehicles: Solutions and challenges. IEEE Internet Things J..

[69] Czech, P., Turoń, K. & Barcik, J. Autonomous vehicles: Basic issues. *Zeszyty Naukowe. Transport/Politechnika Śląska* (2018).

[70] Rozhkova, N., Rozhkova, D. & Blinova, U. in *International Conference on Integrated Science.* 313–324 (Springer).

[71] Jenssen, G. D., Moen, T. & Johnsen, S. O. in *Proceedings of the 26th ITS World Congress, Singapore.* 21–25.

[72] Rozenblit L, Keil F (2002). The misunderstood limits of folk science: An illusion of explanatory depth. Cognit. Sci..

[73] Fox CR, Tversky A (1995). Ambiguity aversion and comparative ignorance. Qual. J. Eng. Econ..

[74] Kruger J, Dunning D (1999). Unskilled and unaware of it: How difficulties in recognizing one's own incompetence lead to inflated self-assessments. J. Pers. Soc. Psychol..

[75] Zhao X, Yang J, Tan H (2022). The effects of subjective knowledge on the acceptance of fully autonomous vehicles depend on individual levels of trust.

[76] Maestre-Andrés S, Drews S, Savin I, van den Bergh J (2021). Carbon tax acceptability with information provision and mixed revenue uses. Nat. Commun..

[77] Lajunen T, Sullman MJ, Gaygısız E (2022). Self-assessed driving skills and risky driver behaviour among young drivers: A cross-sectional study. Front. Psychol..

[78] Shaaban, K. Impact of experience and training on traffic knowledge of young drivers. *Open Transp. J.* **15** (2021).

[79] Feick, L., Park, C. W. & Mothersbaugh, D. L. Knowledge and knowledge of knowledge: What we know, what we think we know, and why the difference makes a difference. *ACR North Am. Adv.* (1992).

[80] Raju PS, Mangold S (1995). Differential effects of subjective knowledge, objective knowledge, and usage experience on decision making: An exploratory investigation. J. Consum. Psychol..

[81] Cole CA, Gaeth G, Chakraborty G, Levin I (1992). Exploring the relationships among self-reported knowledge, objective knowledge, product usage, and consumer decision making. Adv. Consum. Res..

[82] Ranney MA, Clark D (2016). Climate change conceptual change: Scientific information can transform attitudes. Top. Cogn. Sci..

