# Supplementary material for: Knowledge as a key determinant of public support for autonomous vehicles
Source: Sci Rep. 2024 Jan 25;14:2156. doi: 10.1038/s41598-024-52103-6 (PMC10810904; doi:10.1038/s41598-024-52103-6)
Supplement: Supplementary file 1 — Supplementary Information. [file 41598_2024_52103_MOESM1_ESM.pdf]

Supporting information for

Knowledge as a key determinant of public support for autonomous vehicles

Hao Tan[1*†], Jiayan Liu[1*†], Cong Chen[1†], Xue Zhao[1], Jialuo Yang[1], Chao Tang[1]

Correspondence to: htan@hnu.edu.cn; liujiayan@hnu.edu.cn

This PDF file includes:

Materials and Methods
Note S1. Full informational text materials of Studies 1 and. 2
Note S2. Full informational text materials of Study 3a
Note S3. Full informational text materials of Study 3b
Table S1. Sample details for different studies
Table S2. Analysis of linear regression by study
Table S3. The difference of objective knowledge by different levels of support in Study 2a and Study 2b
Table S4. Sensitivity analysis. Regression analysis of public support and scientific literacy after removing the AV question from the "scientific literacy" scale
References

Materials and Methods

Data

Online respondents were recruited through Baidu Data Crowdsourcing Platform, with more than 17,000,000 respondents in its sample database and covering 300 cities in China. For the online studies, we aimed to collect data about 1000 participants per study. However, there is some uncertainty about the number of people that the survey company would be able to recruit, and that we could end up with slightly different sample sizes for each study. At the end of data collection, the survey company recruited 1052, 938, 1010, and 817 participants in Study 1, Study 2a, Study 2b, and Study 3b, respectively. Due to the difficulty of recruiting for offline experiments, our researcher recruited participants over the age of 18 a s possible, resulting in 357 participants. Quality control of the questionnaire was done by checking the time of participation, and 62 non-compliant participants were excluded, which resulted in a total of 4112 participants included in our final study analysis. The field experiment was conducted from January 16 to March 6, 2020. We arranged 33 trained researchers from the experimental team to conduct respondent recruitment and interviews across China. The survey company will pay participants a compensation if they complete the questionnaires. Participants who complete the offline experiment will receive a ¥20 reward at the end of the experiment.

Instrument development

The scientific literacy (objective knowledge about science) measure construction in Study1 is adopted from previous research about scientific knowledge[1,2], which is widely used in surveys of public knowledge in various countries[3,4]. The measure items are considered elementary nature, including the public's knowledge of physics and earth sciences(5 items), astronomy(2 items), biologic sciences and human origins(2 items), health care(3 items), and AV(1 item). These 15 true-false questions measured respondents' scientific literacy score, which was calculated as a total score based on the results of their answers (1 point for the correct answer, 0 points for the wrong answer, or "Do not know"). Having a high level of scientific literacy means having a good understanding of the process of scientific study, disciplinary constructs and some contemporary political issues[5]. Similar to other studies on scientific knowledge[6], one item in the "scientific literacy" scale refer to our research topic(AV). For robustness, we also replicated the analyses after removing the AV question from the "scientific literacy" scale. The results of the sensitivity analysis are consistent with our reported conclusions, which indicates that our results are robust. Details are shown in Table S4.

Self-assessed knowledge of AVs. The construct of self-assessed knowledge adopted from previous research[7-10], with Cronbach's alpha values of 0.864, 0.862 and 0.890 for the 7-point, 9-point and 100-point scales, respectively.

Objective knowledge about AVs. The development of measures for the timely study of emerging phenomena such as automated vehicles do not always permit careful psychometric testing[11], and there hasn't been a universally-acknowledged scientific paradigm measuring objective knowledge to date[10]. Therefore, with reference of the National Science Foundation's measure of the public's scientific literacy, we divide the measure of AV objective knowledge into three dimensions: understanding the definitions of AV (items 1-3), understanding AV-related concepts (e.g., terms such as degree of automation, connected vehicles, etc.) (items 4-7), and understanding of some public policy issues or social conditions that involve or directly affect AV (items 8-10).

Public support for AVs. The public support was measured by asking respondents what extent they support for AVs, ranging from “not support at all” to “very support” .This single measure was adapted from previous studies[2,12-15]. We also tested the reliability of the construct of support, a 3-item survey with a Cronbach's alpha of α = 0.781.

Methods

The design of our questionnaire mainly includes the following steps:

1. To learn more comprehensively and profoundly about the relationship between individual knowledge and support for AVs, we identified the following main research points:

1) The relationship between public’s self-assessed and objective knowledge and support for AVs;

2) The relationship between public’s self-assessed and objective knowledge and willingness to ride and buy AVs;

3) The relationship between public’s self-assessed and objective knowledge and the scenarios of different risk holders related to AVs;

4) Impact of changes in public’s self-assessed or objective knowledge on support for AVs;

2. We tested and evaluated preliminary questionnaires on the Baidu Data Crowdsourcing Platform.

3. We adhered to the Baidu Data Crowdsourcing Platform’s requirements and modified the four questionnaires’ format and content, reducing each study’s time to 5–10 minutes.

4. We divided the research into two parts: the online survey Study 1, 2, and 3b were completed through the Baidu Data Crowdsourcing Platform; Study 3a was completed through the experiment because it required researchers to provide respondents with feedback on their real knowledge scores.

After a brief experimental introduction, the respondents were asked whether they would accept and complete the survey. Subsequently, we first asked the respondents about their access to information about AVs and then explained the AVs (the explanation is based on SAE J3016’s definition of Level 4 and Level 5 autonomous driving systems).

In all studies (N=4112), we collected data on the support for AVs (Study 1, Study 3a, Study 3b: support for AVs; Study 2a: support for AVs, willingness to ride and buy AVs; Study 2 b: support for AVs with the scenarios of different risk holders) and their self-assessed knowledge as well as objective knowledge measured by 10–15 questions.

Additional questions were to measure the change of respondents’ self-assessed and objective knowledge in Study 3a and Study 3b. Questions about demographic information were at the end of each questionnaire.

Baidu Data Crowdsourcing Platform will provide remuneration for respondents who complete the questionnaire and collect their completion time and data results. The complete questionnaire content can be found in the supplementary materials.

Study 1 is aimed to explore the relationship between the public’s self-assessed and general objective knowledge and their support for AVs.

First, we asked the respondents to evaluate their support for AVs (“1 = not support at all” and “5 = very support”) and understanding ((“1 = not understand at all” and “5 = understand very well”) using a five-point Likert scale. After completing the assessment, we used 15 true-false questions to measure respondents’ general objective knowledge score, which was calculated by the results of the answers (1 point for the correct answer, 0 points for the wrong answer, or “Do not know”). These questions were derived from previous studies on the public’s knowledge[1,3,16],

including a question about AVs[17]. These questions paved the way for further study of the public's objective knowledge of AVs in subsequent experiments. Finally, we asked the respondents about their basic personal information, including gender, age, income, education, purchasing, and driving experience.

Therefore, Study 2a aims to explore whether the public is willing to ride or buy AVs based on Study 1.

In addition to the "Support for AVs" section of the questionnaire, questions about riding willingness and purchasing willingness ("1 = certainly would not" and "7 = certainly would") were added based on Study 1. We also changed the original 15 questions about general objective knowledge to 10 questions related to AVs, which were adapted from previous studies in the field of AVs[5,17-19].

Study 2b used the form of "situation introduction" to ask respondents to substitute themselves into three different scenarios. The purpose is to explore the public's support for AVs from the scenario of varying risk holders.

Therefore, three scenarios were added to the part of "Support for AVs" in a questionnaire. Further, the respondents were asked about their support for AVs from these scenarios ("1 = not support at all" and "9 = very support"). The descriptions of these scenarios are as follows: "Please imagine yourself riding in AVs in the future"; "Please imagine your relatives or friends riding in AVs in the future"; and "Please imagine AVs driving on the road in the future."

Study 3a aims to explore how changes in respondents' self-assessed knowledge affects their support for AVs through experiments.

Similar to previous research, we first asked respondents to evaluate their support and understanding of AVs on a 100-point scale. The aim of measurement change is to analyze respondents' change of support and self-assessed understanding after self-assessed knowledge changes. Subsequently, we measured respondents' objective knowledge in the field of AVs using the same 10 questions as in Study 2.

We changed respondents' self-assessed knowledge by showing them their score in the objective knowledge test, reflecting their real self-assessed knowledge. After answering 10 true-false questions about AVs' objective knowledge, the test score was provided to the respondents by the researchers. Subsequently, respondents were asked to evaluate how much they knew about AVs again. Finally, the respondents were asked to choose a value from 1–100 to assess their support for AVs and fill in the basic personal information.

In Study 3b, we used the same research strategy to explore how objective knowledge changes affected AVs' support.

This study's process was similar to Study 3a, except that after collecting the initial data of respondents' knowledge and support, we changed the respondents' objective knowledge and collected the changes of support. Respondents were asked to read three paragraphs of text about AVs. Based on the results of previous research, the text was divided into three aspects: research history, concept, and definition, as well as relevant laws and regulations. After the reading, the respondents were asked to answer three questions based on what they had read, which was regarded as measuring whether their objective knowledge had changed.

Note S1. Full informational text materials of Studies 1 and 2

(i) Study 1 Survey Question

Introduction to Survey:

This questionnaire will ask you about your knowledge and support for autonomous vehicles (hereafter referred to as AVs), and some information that is not personal privacy. The questionnaire is expected to take you 5-10 minutes. We would appreciate your participation.

If you do not have enough knowledge about the relevant content in the questionnaire, or this does not currently belong to your research field, it does not matter. We hope to get your real views and opinions, which will be very helpful for our research.

- Explanation of AVs:

AVs, also known as driverless vehicles, self-driving cars. It uses a driverless system to complete all driving operations, and human drivers can take over.

- AVs Support Questions:

"Please rate your level of support for AVs." ("1 = not support at all" and "5 = strongly support")

- AVs Self-assessed Knowledge Questions:

"Please rate your understanding of AVs." ("1 = not understand at all" and "5 = understand very well")

- AVs Objective Knowledge Questions

1. The center of the Earth is very hot. (True, False, I don't know)

2. The continents have been moving their location for millions of years and will continue to move. (True, False, I don't know)

3. The earth orbits the sun. (True, False, I don't know)

4. All radioactivity is man-made. (True, False, I don't know)

5. Electrons are smaller than atoms. (True, False, I don't know

6. Lasers work by focusing sound waves. (True, False, I don't know)

7. The universe began with a huge explosion. (True, False, I don't know)

8. It is the father's gene that decides whether the baby is a boy or a girl. (True, False, I don't know)

9. Antibiotics kill viruses as well as bacteria. (True, False, I don't know)

10. Human beings, as we know them today, developed from earlier species of animals. (True, False, I don't know)

11. The oxygen we breathe comes from plants. (True, False, I don't know)

12. The earliest humans lived at the same time as dinosaurs. (True, False, I don't know)

13. Smoking may cause lung cancer. (True, False, I don't know)

14. Sunlight may cause skin cancer. (True, False, I don't know)

15. Autonomous vehicles are equivalent to connected cars. (True, False, I don't know)

- Demographics:

"What is your gender?"

Male, Female

"What is your age?"

18-29, 30-44, 45-59, 60 and above

"What is the highest level of education you have received?"

Middle school and below, High school, Junior college, Undergraduate, Graduate and above

"What is your personal monthly income? (CNY)"

Below 2000, 2000-5000, 5000-10000, More than 10000

"Do you have any experience driving a car on an open road?"

Yes, No

"What is your driving age (calculated from the time you get your driving license)?"

No driving license, Within 1 year, 1-3 years, 3-5 years, 5-10 years, 10 years or more

"What is your main way of commuting?"

Walking and cycling, Driving or taking a car (small motor vehicle with less than seven seats), Public transportation (Bus & subway), Other

"How much time do you drive or ride in cars (small motor vehicles with less than seven seats) per day?"

Less than 0.5 hours, 0.5-1 hour, 1-2 hours, More than 2 hours

"Do you or your family own your own car (small motor vehicle with less than seven seats)?"

Yes, No

"Does your current car have any autonomous driving functions?"

Yes, No, I don't know

<u>(ii) Study 2a Survey Question</u>

Introduction to Survey:

This questionnaire will ask you about your knowledge and support for autonomous vehicles (hereafter referred to as AVs), and some information that is not personal privacy. The questionnaire is expected to take you 5-10 minutes. We would appreciate your participation.

If you do not have enough knowledge about the relevant content in the questionnaire, or this does not currently belong to your research field, it does not matter. We hope to get your real views and opinions, which will be very helpful for our research.

- Explanation of AVs:

AVs, also known as driverless vehicles, self-driving cars. It uses a driverless system to complete all driving operations, and human drivers can take over.

- AVs Support Questions:

"Please rate your level of support for AVs." ("1 = not support at all" and "7 = strongly support")

"Are you willing to ride an AV?" ("1 = certainly would not" and "7 = certainly would ")

"Are you willing to buy an AV?" ("1 = certainly would not" and "7 = certainly would ")

- AVs Self-assessed Knowledge Questions:

"Please rate your understanding of AVs." ("1 = not understand at all" and "7 = understand very well")

"Among the people I know, I can be regarded as an "expert" in the field of AVs." ("1 = strongly disagree" and "7 = strongly agree")

"I know how to identify and pick AVs." ("1 = strongly disagree" and "7 = strongly agree")

- AVs Objective Knowledge Questions

1. AVs can drive without human control. (True, False, I don't know)

2. AVs are cars with driver assistance technology. (True, False, I don't know)

3. An AV is a car driven by a robot. (True, False, I don't know)

4. In the minimum degree of automation of AVs, it can complete all driving tasks in any scenario. (True, False, I don't know)

5. AVs have the same meaning as connected cars, so the two terms are used interchangeably. (True, False, I don't know)

6. Cars can be interconnected without the need for autonomous driving technology. (True, False, I don't know)

7. An AV can run with different degrees of automation. (True, False, I don't know)

8. The first AV appeared in 1920. (True, False, I don't know)

9. Companies in China have begun testing AVs. (True, False, I don't know)

10. So far, AVs have caused deaths. (True, False, I don't know)

- Demographics:

  "What is your gender?"

  Male, Female

  "What is your age?"

  18-29, 30-44, 45-59, 60 and above

  "What is the highest level of education you have received?"

  Middle school and below, High school, Junior college, Undergraduate, Graduate and above

  "What is your personal monthly income? (CNY)"

  Below 2000, 2000-5000, 5000-10000, More than 10000

  "Do you have any experience driving a car on an open road?"

  Yes, No

  "What is your driving age (calculated from the time you get your driving license)?"

  No driving license, Within 1 year, 1-3 years, 3-5 years, 5-10 years, 10 years or more

  "What is your main way of commuting?"

  Walking and cycling, Driving or taking a car (small motor vehicle with less than seven seats), Public transportation (Bus & subway), Other

  "How much time do you drive or ride in cars (small motor vehicles with less than seven seats) per day?"

  Less than 0.5 hours, 0.5-1 hour, 1-2 hours, More than 2 hours

  "Do you or your family own your own car (small motor vehicle with less than seven seats)?"

  Yes, No

  "Does your current car have any autonomous driving functions?"

  Yes, No, I don't know

<u>(iii) Study 2b Survey Question</u>

- Introduction to Survey:

  This questionnaire will ask you about your knowledge and support for autonomous vehicles (hereafter referred to as AVs), and some information that is not personal privacy. The questionnaire is expected to take you 5-10 minutes. We would appreciate your participation.

  If you do not have enough knowledge about the relevant content in the questionnaire, or this does not currently belong to your research field, it does not matter. We hope to get your real views and opinions, which will be very helpful for our research.

- Explanation of AVs:

  AVs, also known as driverless vehicles, self-driving cars. It uses a driverless system to complete all driving operations, and human drivers can take over.

- AVs Support Questions:

Scenario 1

Please imagine yourself riding in AVs in the future.

"Please rate your level of support for AVs." ("1 = not support at all" and "9 = strongly support")

Scenario 2

Please imagine your relatives or friends riding in AVs in the future.

"Please rate your level of support for AVs." ("1 = not support at all" and "9 = strongly support")

Scenario 3

Please imagine AVs driving on open roads in the future.

"Please rate your level of support for AVs." ("1 = not support at all" and "9 = strongly support")

- AVs Self-assessed Knowledge Questions:

"Please rate your understanding of AVs." ("1 = not understand at all" and "9 = understand very well")

"Among the people I know, I can be regarded as an "expert" in the field of AVs." ("1 = strongly disagree" and "9 = strongly agree")

"I know how to identify and pick AVs." ("1 = strongly disagree" and "9 = strongly agree")

- AVs Objective Knowledge Questions:

1. AVs can drive without human control. (True, False, I don't know)

2. AVs are cars with driver assistance technology. (True, False, I don't know)

3. An AV is a car driven by a robot. (True, False, I don't know)

4. In the minimum degree of automation of AVs, it can complete all driving tasks in any scenario. (True, False, I don't know)

5. AVs have the same meaning as connected cars, so the two terms are used interchangeably. (True, False, I don't know)

6. Cars can be interconnected without the need for autonomous driving technology. (True, False, I don't know)

7. An AV can run with different degrees of automation. (True, False, I don't know)

8. The first AV appeared in 1920. (True, False, I don't know)

9. Companies in China have begun testing AVs. (True, False, I don't know)

10. So far, AVs have caused deaths. (True, False, I don't know)

- Demographics:

"What is your gender?"

Male, Female

"What is your age?"

18-29, 30-44, 45-59, 60 and above

"What is the highest level of education you have received?"

Middle school and below, High school, Junior college, Undergraduate, Graduate and above

"What is your personal monthly income? (CNY)"

Below 2000, 2000-5000, 5000-10000, More than 10000

"Do you have any experience driving a car on an open road?"

Yes, No

"What is your driving age (calculated from the time you get your driving license)?"

No driving license, Within 1 year, 1-3 years, 3-5 years, 5-10 years, 10 years or more

"What is your main way of commuting?"

Walking and cycling, Driving or taking a car (small motor vehicle with less than seven seats), Public transportation (Bus & subway), Other

"How much time do you drive or ride in cars (small motor vehicles with less than seven seats) per day?"

Less than 0.5 hours, 0.5-1 hour, 1-2 hours, More than 2 hours

"Do you or your family own your own car (small motor vehicle with less than seven seats)?"

Yes, No

"Does your current car have any autonomous driving functions?"

Yes, No, I don't know

Note S2. Full informational text materials of Study 3a.

<u>(i) Study 3a Survey Question</u>

<u>Part 1</u>

- Introduction to Survey:

The following survey is part of a research project carried out by State Key Laboratory of Advanced Design and Manufacturing for Vehicle Body, which is about your knowledge and support for autonomous vehicles (hereafter referred to as AVs), and some information that is not personal privacy Our research results will be used to explore public's attitude and behaviors towards the development of science and technology in the future.

If you do not have enough knowledge about the relevant content in the questionnaire, or this does not currently belong to your research field, it does not matter. We hope to get your real views and opinions, which will be very helpful for our research.

The following content has been approved by the Ethics Committee of School of Design of Hunan University.

- Explanation of AVs:

AVs, also known as driverless vehicles, self-driving cars. It uses a driverless system to complete all driving operations, and human drivers can take over.

- AVs Support Questions:

"Please rate your level of support for AVs." ("1 = not support at all" and "100 = strongly support ")

- AVs Self-assessed Knowledge Questions:

"Please rate your understanding of AVs." ("1 = not understand at all" and "100 = understand very well")

"Among the people I know, I can be regarded as an "expert" in the field of AVs." ("1 = strongly disagree" and "100 = strongly agree")

"I know how to identify and pick AVs." ("1 = strongly disagree" and "100 = strongly agree")

- AVs Objective Knowledge Questions:

1. AVs can drive without human control. (True, False, I don't know)

2. AVs are cars with driver assistance technology. (True, False, I don't know)

3. An AV is a car driven by a robot. (True, False, I don't know)

4. In the minimum degree of automation of AVs, it can complete all driving tasks in any scenario. (True, False, I don't know)

5. AVs have the same meaning as connected cars, so the two terms are used interchangeably. (True, False, I don't know)

6. Cars can be interconnected without the need for autonomous driving technology. (True, False, I don't know)

7. An AV can run with different degrees of automation. (True, False, I don't know)

8. The first AV appeared in 1920. (True, False, I don't know)

9. Companies in China have begun testing AVs. (True, False, I don't know)

10. So far, AVs have caused deaths. (True, False, I don't know)

Part 2

- AVs Support Questions:

"Please rate your level of support for AVs." ("1 = not support at all" and "100 = strongly support ")

- AVs Self-assessed Knowledge Questions:

"Please rate your understanding of AVs." ("1 = not understand at all" and "100 = understand very well")

- Demographics:

"What is your gender?"
Male, Female
"What is your age?"
18-29, 30-44, 45-59, 60 and above
"What is the highest level of education you have received?"
Middle school and below, High school, Junior college, Undergraduate, Graduate and above
"What is your personal monthly income? (CNY)"
Below 2000, 2000-5000, 5000-10000, More than 10000
"Do you have any experience driving a car on an open road?"
Yes, No
"What is your driving age (calculated from the time you get your driving license)?"
No driving license, Within 1 year, 1-3 years, 3-5 years, 5-10 years, 10 years or more
"What is your main way of commuting?"
Walking and cycling, Driving or taking a car (small motor vehicle with less than seven seats), Public transportation (Bus & subway), Other
"How much time do you drive or ride in cars (small motor vehicles with less than seven seats) per day?"
Less than 0.5 hours, 0.5-1 hour, 1-2 hours, More than 2 hours
"Do you or your family own your own car (small motor vehicle with less than seven seats)?"
Yes, No
"Does your current car have any autonomous driving functions?"
Yes, No, I don't know

(ii) Study 3a Field experiment Process

We arranged 33 trained researchers from the experimental team to conduct respondent recruitment and interviews across China. We paid a fee to researchers and respondents. A total of

357 respondents from across the country were recruited, excluding those who refused to be interviewed. The overall purpose of the study was to explore whether the change of self-assessed knowledge can influence public support for AVs.

- Conduct self-introduction:

"Hi! My name is XX and I 'm a researcher at Hunan University. We are conducting new research about public's knowledge and support for autonomous vehicles (hereafter referred to as AVs)."

- Questionnaire process arrangement:

1. Read and complete informed consent forms: Firstly, participants were required to read the relevant instructions in the questionnaire and confirm whether they were willing to participate in our research;

2. In Part 1, participants were asked to show their support and knowledge of AVs, the content and order of the inquiry strictly followed the content of the questionnaire, the time of which was controlled within 10 minutes.

The researcher could answer the questions of the participant's understanding of the questionnaire, but the questions about AVs knowledge would not be answered.

3. The researcher recovered the answer of "Part 1" and calculates participant's objective knowledge score.

4. Participants were informed their objective knowledge score, and didn't get explanation of the results at this stage. But participants could get explanation after the survey if they were interested.

Participants were asked to complete Part 2, the time of which was controlled within 5 minutes.

Note S3. Full informational text materials of Study 3b

- Introduction to Survey:

This questionnaire will ask you about your knowledge and support for autonomous vehicles (hereafter referred to as AVs), and some information that is not personal privacy. The questionnaire is expected to take you 5-10 minutes. We would appreciate your participation.

If you do not have enough knowledge about the relevant content in the questionnaire, or this does not currently belong to your research field, it does not matter. We hope to get your real views and opinions, which will be very helpful for our research.

- Explanation of AVs:

AVs, also known as driverless vehicles, self-driving cars. It uses a driverless system to complete all driving operations, and human drivers can take over.

- AVs Support Questions:

"Please rate your level of support for AVs." ("1 = not support at all" and "100 = strongly support ")

- AVs Self-assessed Knowledge Questions:

"Please rate your understanding of AVs." ("1 = not understand at all" and "100 = understand very well")

"Among the people I know, I can be regarded as an "expert" in the field of AVs." ("1 = strongly disagree" and "100 = strongly agree")

“I know how to identify and pick AVs.” ("1 = strongly disagree" and "100 = strongly agree")

- AVs Objective Knowledge Questions:

1. AVs can drive without human control. (True, False, I don’t know)

2. AVs are cars with driver assistance technology. (True, False, I don’t know)

3. An AV is a car driven by a robot. (True, False, I don’t know)

4. In the minimum degree of automation of AVs, it can complete all driving tasks in any scenario. (True, False, I don’t know)

5. AVs have the same meaning as connected cars, so the two terms are used interchangeably. (True, False, I don’t know)

6. Cars can be interconnected without the need for autonomous driving technology. (True, False, I don’t know)

7. An AV can run with different degrees of automation. (True, False, I don’t know)

8. The first AV appeared in 1920. (True, False, I don’t know)

9. Companies in China have begun testing AVs. (True, False, I don’t know)

10. So far, AVs have caused deaths. (True, False, I don’t know)

- Reading Materials

Section One

The first batch of real AVs appeared in the 1980s, and then universities and companies successively carried out research and testing of AVs, and in 1995 completed the first autonomous coast-to-coast drive of the United States. In 2017, Waymo announced that it had begun testing AVs, however, there was still an employee in the car. In October 2018, Waymo announced that its test vehicles had traveled in automated mode for over 10,000,000 miles (16,000,000 km).

Section Two

AVs are vehicles that can sense the environment and drive safely with little or no human intervention. The premise of AVs driving on public roads is to meet the following three conditions:

(1) Has a manual override feature that allows a driver to assume control of the AV at any time;

(2) Has a driver seated in the control seat of the vehicle while in operation who is prepared to take control of the AV at any moment;

(3) Has ability to comply with traffic laws, motor vehicle laws and traffic control devices specified in the driving area.

Modern vehicles provide partly automated features, namely driver assistance technology, but there are still differences between AVs and driver assistance technology.

Section Three

In June 2011, Nevada in the United States became the first jurisdiction in the world to legally operate AVs on public roads. In 2013, the British government allowed testing of AVs on public roads. In 2014, the French government announced that it would allow AVs to be tested on public roads in 2015. As of 2019, 29 states in the U.S. have passed laws allowing AVs.

- AVs Objective Knowledge Questions after Reading

“Waymo announced that it had begun testing AVs without a safe driver, with no one on board.” (True, False, I don’t remember)

“Fully automatic driving technology, namely driver assistance technology.” (True, False, I don’t remember)

"As of 2019, 29 states in the U.S. have passed laws allowing AVs." (True, False, I don't remember)

- AVs Support Questions after Reading:

"Please rate your level of support for AVs after reading." ("1 = not support at all" and "100 = strongly support")

- Demographics:

"What is your gender?"
Male, Female
"What is your age?"
18-29, 30-44, 45-59, 60 and above
"What is the highest level of education you have received?"
Middle school and below, High school, Junior college, Undergraduate, Graduate and above
"What is your personal monthly income? (CNY)"
Below 2000, 2000-5000, 5000-10000, More than 10000
"Do you have any experience driving a car on an open road?"
Yes, No
"What is your driving age (calculated from the time you get your driving license)?"
No driving license, Within 1 year, 1-3 years, 3-5 years, 5-10 years, 10 years or more
"What is your main way of commuting?"
Walking and cycling, Driving or taking a car (small motor vehicle with less than seven seats), Public transportation (Bus & subway), Other
"How much time do you drive or ride in cars (small motor vehicles with less than seven seats) per day?"
Less than 0.5 hours, 0.5-1 hour, 1-2 hours, More than 2 hours
"Do you or your family own your own car (small motor vehicle with less than seven seats)?"
Yes, No
"Does your current car have any autonomous driving functions?"
Yes, No, I don't know

Table S1. Sample details for different studies

Table S1a. Demographic characteristic variables of Study 1 respondents

Demographic characteristic variables		N	Percentage / %
**Gender**	Male	523	50.4
	Female	514	49.6
**Age**	18 - 29	590	56.9
	30 - 44	350	33.8
	45 - 59	70	6.8
	≥ 60	27	2.6
**Education**	Middle school and below	94	9.1
	High school	147	14.2
	Junior college	173	16.7

	Undergraduate	499	48.1
	Graduate and above	124	12.0
**Monthly Income (CNY)**	< 2000	272	26.2
	2000 - 5000	367	35.4
	5000 - 10000	270	26.0
	> 10000	128	12.3
**Driving Experience**	Yes	669	64.5
	No	368	35.5
**License / year**	No	303	29.2
	< 1	160	15.4
	1-3	231	22.3
	3-5	135	13.0
	5-10	137	13.2
	> 10	71	6.8
**Commute**	Walking and cycling	242	23.3
	Driving or taking a car	429	41.4
	Public transportation	310	29.9
	Other	56	5.4
**Driving or Riding Time**	< 0.5 hour	547	52.7
	0.5 - 1 hour	333	32.1
	1 - 2 hours	119	11.5
	> 2 hours	38	3.7
**Car owner**	Yes	771	74.3
	No	266	25.7
**Autonomous functions**	Yes	206	19.9
	No	184	75.6
	I don’t know	47	4.5

Table S1b. Demographic characteristic variables of Study 2a respondents

**Demographic characteristic variables**		**N**	**Percentage / %**
**Gender**	Male	438	47.6
	Female	477	52.1
**Age**	18 - 29	579	63.3
	30 - 44	269	29.4
	45 - 59	54	5.9
	≥ 60	13	1.4
**Education**	Middle school and below	48	5.2
	High school	119	13.0
	Junior college	159	17.4
	Undergraduate	471	51.5
	Graduate and above	118	12.9
**Monthly Income (CNY)**	< 2000	240	26.2

	2000 - 5000	309	33.8
	5000 - 10000	266	29.1
	> 10000	100	10.9
**Driving Experience**	Yes	548	59.9
	No	367	40.1
**License / year**	No	302	33.0
	< 1	116	12.7
	1-3	199	21.7
	3-5	137	15.0
	5-10	113	12.3
	> 10	48	5.2
**Commute**	Walking and cycling	195	21.3
	Driving or taking a car	363	39.7
	Public transportation	315	34.4
	Other	42	4.6
**Driving or Riding Time**	< 0.5 hour	472	51.6
	0.5 - 1 hour	307	33.6
	1 - 2 hours	103	11.3
	> 2 hours	33	3.6
**Car owner**	Yes	683	74.6
	No	232	25.4
**Autonomous functions**	Yes	177	19.3
	No	683	74.6
	I don’t know	55	6.0

Table S1c. Demographic characteristic variables of Study 2b respondents

**Demographic characteristic variables**		**N**	**Percentage / %**
**Gender**	Male	495	49.7
	Female	500	50.3
**Age**	18 - 29	569	57.2
	30 - 44	345	34.7
	45 - 59	65	6.5
	≥ 60	16	1.6
**Education**	Middle school and below	73	7.3
	High school	188	18.9
	Junior college	188	18.9
	Undergraduate	463	46.5
	Graduate and above	83	8.3
**Monthly Income (CNY)**	< 2000	177	17.8
	2000 - 5000	373	37.5
	5000 - 10000	352	35.4
	> 10000	93	9.3
**Driving Experience**	Yes	670	67.3

	No	325	32.7
**License / year**	No	249	25.0
	< 1	127	12.8
	1-3	264	26.5
	3-5	190	19.1
	5-10	106	10.7
	> 10	59	5.9
**Commute**	Walking and cycling	195	19.6
	Driving or taking a car	516	51.9
	Public transportation	253	25.4
	Other	31	3.1
**Driving or Riding Time**	< 0.5 hour	401	40.3
	0.5 - 1 hour	395	39.7
	1 - 2 hours	148	14.9
	> 2 hours	51	5.1
**Car owner**	Yes	786	79.0
	No	209	21.0
**Autonomous functions**	Yes	220	22.1
	No	715	71.9
	I don’t know	60	6.0

Table S1d. Demographic characteristic variables of Study 3a respondents

**Demographic characteristic variables**		**N**	**Percentage / %**
**Gender**	Male	188	52.7
	Female	169	47.3
**Age**	18 - 29	215	60.2
	30 - 44	56	15.7
	45 - 59	76	21.3
	≥ 60	10	2.8
**Education**	Middle school and below	36	10.1
	High school	43	12.1
	Junior college	52	14.6
	Undergraduate	132	37.1
	Graduate and above	93	26.1
**Monthly Income (CNY)**	< 2000	127	35.6
	2000 - 5000	92	25.8
	5000 - 10000	96	26.9
	> 10000	42	11.8
**Driving Experience**	Yes	224	62.7
	No	133	37.3
**License / year**	< 1	186	52.1
	1-3	46	12.9

	3-5	49	13.7
	5-10	40	11.2
	> 10	36	10.1
**Commute**	Walking and cycling	112	31.4
	Driving or taking a car	96	26.9
	Public transportation	136	38.1
	Other	13	3.6
**Driving or Riding Time**	< 0.5 hour	262	73.4
	0.5 - 1 hour	63	17.6
	1 - 2 hours	23	6.4
	> 2 hours	9	2.5
**Car owner**	Yes	261	73.1
	No	96	26.9
**Autonomous functions**	Yes	45	12.6
	No	284	79.6
	I don't know	28	7.8

Table.S1e Demographic characteristic variables of Study 3b respondents

**Demographic characteristic variables**		**N**	**Percentage / %**
**Gender**	Male	430	53.2
	Female	378	46.8
**Age**	18 - 29	458	56.7
	30 - 44	244	30.2
	45 - 59	94	11.6
	⩾ 60	12	1.5
**Education**	Middle school and below	48	5.9
	High school	101	12.5
	Junior college	82	10.1
	Undergraduate	402	49.8
	Graduate and above	175	21.7
**Monthly Income (CNY)**	< 2000	191	23.6
	2000 - 5000	233	28.8
	5000 - 10000	244	30.2
	> 10000	140	17.3
**Driving Experience**	Yes	531	65.7
	No	277	34.3
**License / year**	No	201	24.9
	< 1	84	10.4
	1-3	153	18.9
	3-5	157	19.4
	5-10	131	16.2
	> 10	82	10.1

**Commute**	Walking and cycling	152	18.8
	Driving or taking a car	349	43.2
	Public transportation	271	33.5
	Other	36	4.5
**Driving or Riding Time**	< 0.5 hour	407	50.4
	0.5 - 1 hour	258	31.9
	1 - 2 hours	106	13.1
	> 2 hours	37	4.6
**Car owner**	Yes	608	75.2
	No	200	24.8
**Autonomous functions**	Yes	136	16.8
	No	615	76.1
	I don’t know	57	7.1

Table S2. Analysis of linear regressions by studies

Table S2a. Effects of self-assessed knowledge on support for AVs.

	Support						
	Study1	Study2a	Study2b (scenario 1)	Study2b (scenario 2)	Study2b (scenario 3)	Study3a	Study3b
**Self-assessed Knowledge**	0.247***	0.227***	0.221***	0.248***	0.304***	0.170***	0.291***
**Constant**	2.985***	3.916***	5.248***	4.971***	4.754***	69.051***	59.203***
**Observations**	1037	915	995	995	995	357	808

Table S2b. Effects of objective knowledge on support for AVs.

	Support						
	Study1	Study2a	Study2b (scenario 1)	Study2b (scenario 2)	Study2b (scenario 3)	Study3a	Study3b
**Objective Knowledge**	0.046***	0.073*	0.074	0.077	0.110*	1.264*	2.413***
**Constant**	3.181***	4.372***	5.875***	5.702***	5.573***	67.061***	57.024***
**Observations**	1037	915	995	995	995	357	808

Note: Respondents reported their support for AVs, self-assessed knowledge and objective knowledge. Knowledge scores for both Study 3a and Study3b are using pre-experimental data. The effects based on linear regression analysis in SPSS applied to all studies. $*P < 0.05$, $**P < 0.01$, $***P < 0.001$.

Table S3. The difference of objective knowledge by different levels of support in Study 2a and Study 2b

	Objective Knowledge			
	Study 2a	Study 2b		
		Scenario 1	Scenario 2	Scenario 3
**Support**	-0.357**	-0.352**	-0.327**	-0.359**
**Constant**	5.387***	5.044***	5.044***	5.051***

Note: Support was chosen as the reference standard, comparing to the other remaining groups by creating dummy variables and linear regression command. *P < 0.05; **P < 0.01; ***P < 0.001.

Table S4. Sensitivity analysis. Regression analysis of public support and scientific literacy after removing the AV question from the "scientific literacy" scale

Coefficients							
Model	Unstandardized Coefficients		Standardized Coefficients			95.0% Confidence Interval for B	
	B	Std. Error	Beta	t	Sig.	Lower Bound	Upper Bound
(Constant)	3.201	0.140		22.876	0.000	2.926	3.475
Scientific literacy	0.046	0.014	0.103	3.338	0.001	0.019	0.073

References

1 Zhang, Z. & Zhang, J. A survey of public scientific literacy in China. *Public understanding of science* **2**, 21 (1993).

2 Besley, J. C., Lee, N. M. & Pressgrove, G. Reassessing the variables used to measure public perceptions of scientists. *Science Communication* **43**, 3-32 (2021).

3 Durant, J. R., Evans, G. A. & Thomas, G. P. The public understanding of science. *Nature* **340**, 11-14 (1989).

4 Arvizu, D. & Bowen, R. National Science Board. *Science and Engineering Indicators 2014* (2014).

5 Miller, J. D. Scientific literacy: A conceptual and empirical review. *Daedalus*, 29-48 (1983).

6 Fernbach, P. M., Light, N., Scott, S. E., Inbar, Y. & Rozin, P. Extreme opponents of genetically modified foods know the least but think they know the most. *Nature Human Behaviour* **3**, 251-256 (2019).

7 Zhao, X., Yang, J. & Tan, H. The Effects of Subjective Knowledge on the Acceptance of Fully Autonomous Vehicles Depend on Individual Levels of Trust. *Springer, Cham* (2022).

8 Aertsens, J., Mondelaers, K., Verbeke, W., Buysse, J. & Huylenbroeck, G. V. The influence of subjective and objective knowledge on attitude, motivations and consumption of organic food. *British food journal* (2011).
9 Brucks, M. The effects of product class knowledge on information search behavior. *Journal of consumer research* **12**, 1-16 (1985).
10 Tan, H., Zhao, X. & Yang, J. Exploring the influence of anxiety, pleasure and subjective knowledge on public acceptance of fully autonomous vehicles. *Computers in Human Behavior* **131**, 107187 (2022).
11 Sanbonmatsu, D. M., Strayer, D. L., Yu, Z., Biondi, F. & Cooper, J. M. Cognitive underpinnings of beliefs and confidence in beliefs about fully automated vehicles. *Transportation research part F: traffic psychology and behaviour* **55**, 114-122 (2018).
12 Jones, M. S., Delborne, J. A., Elsensohn, J., Mitchell, P. D. & Brown, Z. S. Does the US public support using gene drives in agriculture? And what do they want to know? *Science advances* **5**, eaau8462 (2019).
13 Mielby, H., Sandøe, P. & Lassen, J. The role of scientific knowledge in shaping public attitudes to GM technologies. *Public Understanding of Science* **22**, 155-168 (2013).
14 Flores, A. *et al.* Politicians polarize and experts depolarize public support for COVID-19 management policies across countries. *Proceedings of the National Academy of Sciences* **119**, e2117543119 (2022).
15 Kolodinsky, J. & Lusk, J. L. Mandatory labels can improve attitudes toward genetically engineered food. *Science advances* **4**, eaaq1413 (2018).
16 Talebpour, A. & Mahmassani, H. S. Influence of connected and autonomous vehicles on traffic flow stability and throughput. *Transportation Research Part C: Emerging Technologies* **71**, 143-163 (2016).
17 International, S. (SAE International Warrendale, PA, USA, 2014).
18 BBC News. *Tesla in fatal California crash was on Autopilot*, <https://www.bbc.com/news/world-us-canada-43604440> (2018).
19 Lu, N., Cheng, N., Zhang, N., Shen, X. & Mark, J. W. Connected vehicles: Solutions and challenges. *IEEE internet of things journal* **1**, 289-299 (2014).
